# Exposure to maternal vaginal flora during labor and long-term infectious morbidity of the offspring

**DOI:** 10.1007/s00404-025-08240-y

**Published:** 2026-01-06

**Authors:** Ofek Ben-Dahan, Gil Gutvirtz, Tamar Wainstock, Eyal Sheiner

**Affiliations:** 1 Faculty of Health Sciences, Joyce and Irving Goldman Medical School, Ben-Gurion University of the Negev, Beer-Sheva, Israel; 2 Department of Obstetrics and Gynecology, Soroka University Medical Center, Beer-Sheva, Israel; 3 Department of Public Health, Faculty of Health Sciences, Ben-Gurion University of the Negev, Beer-Sheva, Israel

**Keywords:** Maternal vaginal flora, Long-term infectious morbidity, Cesarean delivery, Vaginal delivery

## Abstract

**Purpose:**

Cesarean delivery (CD) has been linked to increased long-term infectious morbidity in offspring, potentially due to limited exposure to the maternal vaginal microbiome, which may influence immune development. We hypothesized that the degree of exposure to vaginal microbiota during labor would be associated with differences in long-term infectious morbidity.

**Methods:**

We conducted a population-based cohort study including 348,332 singleton deliveries. Offspring were classified into four groups: vaginal delivery (VD, reference), CD for non-progressive labor in the first stage (NPL1), CD for non-progressive labor in the second stage (NPL2), and elective (pre-labor) CD. Infectious-related hospitalizations up to age 18 were assessed. Kaplan–Meier curves compared cumulative incidence between the groups and a Cox proportional hazards model adjusted for various potential confounders.

**Results:**

Of the cohort, 89.2% were VD, 1.4% NPL1, 0.6% NPL2, and 8.8% elective CD. Infectious-related hospitalization rates were higher for NPL1 and elective CD (26.2% each) compared to NPL2 (24.3%) and VD (23.8%) (*p* < 0.001). Kaplan–Meier analysis demonstrated a dose–response pattern, with the lowest cumulative incidence in VD, followed by NPL2, NPL1, and highest in elective CD (log-rank *p* < 0.001). In adjusted analysis, NPL1 (aHR 1.10) and elective CD (aHR 1.13) were associated with increased long-term infectious morbidity, whereas NPL2 was not significantly different from VD.

**Conclusion:**

Reduced exposure to vaginal microbiota, as in elective CD and NPL1, is associated with increased long-term infectious morbidity in offspring, while exposure during the second stage of labor (NPL2) may confer immunological benefits.

**Supplementary Information:**

The online version contains supplementary material available at 10.1007/s00404-025-08240-y.

## What does this study add to the clinical work

**Table:** 

Our findings reinforce the potential importance of exposure to maternal vaginal microbiota in reducing long-term infectious morbidity, with vaginal delivery and second-stage intrapartum cesarean delivery demonstrating the lowest risk profiles. These observations may encourage clinicians to consider the immunological value of intrapartum exposure when counseling about delivery mode, particularly in non-urgent scenarios. Nevertheless, as this study is observational and cannot establish causality, interventions aimed at microbial transfer during cesarean delivery—such as vaginal seeding or microbiota transfer—should be approached cautiously. Further evidence from controlled prospective studies is required before routine clinical implementation can be recommended.	

## Introduction

The human microbiota, and specifically the gut microbiota, plays an important role in human health by providing a barrier for colonization of pathogens and by stimulating the development of the immune system [1]. The healthy human fetus is thought to develop within a bacteria-free environment, and the neonatal GI tract is therefore considered a blank canvas to which microbial components are added from birth onward [2]. Exposure to maternal microbes begins at delivery, and the mode of delivery sets the initial pattern of GI tract colonization: infants born vaginally are colonized mainly by microbes from the birth canal and maternal GI tract, whereas infants delivered by cesarean delivery (CD) are initially colonized predominantly by skin flora [3].

Previous studies on children born after elective CD, who lack exposure to maternal vaginal flora, found an increased risk for long-term respiratory [4], cardiovascular [5], neurological [6], and gastrointestinal morbidity [7], as well as higher rates of infectious-related hospitalization in early childhood [8–10]. Immune-related conditions such as asthma [11] and inflammatory bowel disease [12] have also been associated with reduced exposure to the maternal vaginal microbiome. To address this, some researchers studied “vaginal seeding”, exposing neonates to maternal vaginal secretions to compensate for absent exposure during CD. While partial restoration of microbiota was reported [13], the clinical impact remains unclear [14].

As far as we know, the studies conducted on mode of delivery evaluating the risk for future infectious morbidity did not discern between pre-labor elective CD and emergent CD after the onset of labor (intrapartum CD) and did not mention the different timing when intrapartum CD (first or second stage of labor) was undertaken. The second stage of labor begins once full cervical dilation is reached, which, by our hypothesis, fully exposes the fetus to the mother’s vaginal flora and may resemble the exposure occurring during vaginal delivery. In contrast, the first stage of labor, defined as the period before full dilation, provides only limited exposure due to the remaining cervix and physical distance.

Our primary hypothesis was that the mode and stage of delivery are associated with offspring long-term infectious morbidity, with vaginal delivery posing the lowest risk and elective CD the highest. Our secondary hypothesis was that exposure follows a graded pattern, such that second-stage intrapartum CD would approach vaginal delivery in risk, whereas first-stage intrapartum and elective CDs would demonstrate progressively higher morbidity rates.

Hence, we conducted this study to evaluate long-term infectious-related hospitalizations in offspring according to delivery mode, comparing vaginal birth with cesarean deliveries performed at different labor stages as proxies for varying levels of exposure to maternal vaginal flora.

## Methods

This is an extensive population-based cohort analysis including all singleton deliveries that took place between January 1991 and December 2019 in Soroka University Medical Center (SUMC). SUMC is the only hospital in the Negev region, located in the southern district of Israel, with more than 17,000 births annually. The Negev occupies 60% of the land of Israel and is serving the entire population of the region—more than 1,300,000 inhabitants. The study is based on non-selective population data as medical care in Israel is covered by national health insurance law and accessible to all citizens, including deliveries and hospital admissions.

The institutional review board (SUMC-IRB Committee) approved the study that has been performed by the ethical standards laid down in the 1964 Declaration of Helsinki and its later amendments.

We excluded multiple gestations, fetuses with congenital malformations and cases of perinatal mortality from the study. In order to distinguish between the different stages of labor, we also excluded intrapartum complications such as non-reassuring fetal heart rate (NRFHR) tracings, suspected placental abruption, cord prolapse or failed instrumental delivery leading to an emergency CD, which enabled a focus on CDs performed only for the indication of labor dystocia in the first or second stage of labor.

The primary exposure was mode of delivery (VD vs. CD) and the indication for CD. We compared children born via CD for non-progressive labor in the first (NPL1) and second stage (NPL2) and elective CD (pre-labor) with children born vaginally (VD) as a reference group. While the proposed hypothesis is based on the unique definitions of first and second stages of labor and their relation to cervical dilation and fetal proximity to the vaginal flora, it is only a biological assumption and its authentication could not be investigated in the retrospective setting of the study.

Primary outcomes included only the first hospitalizations of the offspring involving any infectious morbidity up to the age of 18 years, as defined by a diagnosis of one of a predetermined list of ICD-9 codes detailed in the supplementary table (Table S1). Follow-up of the offspring was terminated upon the first hospitalization at SUMC involving an infectious illness (i.e., an event) or until censored. Censoring occurred when the subject reached the age of 18 years (by calculation from date of birth), end of the study period (December 2019) or upon death during hospitalization for non-infectious morbidity.

Data were collected from two cross-linked and merged databases available in the SUMC archives: the computerized hospitalization database of SUMC pediatric division that includes demographic information and ICD-9 codes for all medical diagnoses made during a child hospitalizations in SUMC pediatric wards, and the computerized perinatal database of the obstetrics and gynecology department that includes maternal and obstetrical information recorded at admission and immediately following delivery of the mother by an obstetrician. Experienced medical secretaries routinely review the information before entering it into the database to ensure its maximal completeness and accuracy. This way, coding is performed after assessing medical prenatal care records as well as routine hospital documents.

Statistical analysis was performed using SPSS (IBM/ SPSS, Armonk, NY, USA, IBM Corp.) software. Differences in categorical data were assessed by Chi-square for general association, whereas a t-test was used for the comparison of continuous variables with normal distribution. A Kaplan–Meier survival curve was constructed to assess the cumulative infectious-related morbidity in offspring over time in the exposed (CD groups) and unexposed groups (VD group). The log-rank test was used to compare the distributions between the study groups. A Cox proportional hazards model was used to assess a possible independent association between mode of delivery and CD indication with subsequent infectious-related hospitalization risk of the offspring up to 18 years of age while controlling for various confounding factors including maternal age, ethnicity, obesity and smoking, diabetes mellitus and hypertensive disorders, rupture of membranes, gestational age and the child’s birth year. A *p* value of < 0.05 was considered statistically significant.

## Results

A total of 348,332 deliveries met the inclusion criteria: of which 89.2% were vaginal deliveries (VD), 1.4% were CD for NPL1, 0.6% were CD for NPL2, and 8.8% were elective CD**. **Table 1 demonstrates maternal demographic characteristics for all 4 groups. Compared with VD, mothers who had CD (elective or not) were older and more prone to suffer from obesity, hypertensive disorders (chronic hypertension, gestational hypertension or preeclampsia) and diabetes mellitus (pre-gestational or gestational). In addition, they were more likely to have undergone fertility treatments. Nulliparity was twice as frequent in the NPL groups as in VD or elective CD.

**Table 1:** Maternal characteristics of the study population

Characteristic	Vaginal delivery (n = 311,054)	NPL1 (n = 5,149)	NPL2 (n = 2,199)	Elective CD (n = 29,930)	P Value
Maternal age (years, ± SD)	27.9 ± 5.7	29.2 ± 6.0	28.2 ± 5.6	31.1 ± 5.6	< 0.001
Nulliparity, *n* (%)	74,858 (24.1)	2177 (42.3)	1261 (57.3)	3686 (12.3)	< 0.001
Fertility treatments ^a^, *n* (%)	4287 (1.4)	228 (4.4)	72 (3.3)	952 (3.2)	< 0.001
Smoking, *n* (%)	2109 (0.7)	49 (1.0)	10 (0.5)	301 (1.0)	< 0.001
Obesity ^b^, *n* (%)	2750 (0.9)	176 (3.4)	49 (2.2)	910 (3.0)	< 0.001
Ethnicity, Jewish, *n* (%) Bedouin, *n* (%)	139,289 (44.8) 171,765 (55.2)	2,739 (53.2) 2,410 (46.8)	1151 (52.3) 1048 (47.7)	15,056 (50.3) 14,874 (49.7)	< 0.001
Hypertensive disorders ^c^, *n* (%)	12,110 (3.9)	640 (12.4)	191 (8.7)	2325 (7.8)	< 0.001
Diabetes mellitus ^d^, *n* (%)	12,442 (4.0)	530 (10.3)	186 (8.5)	2963 (9.9)	< 0.001

^a^Including all artificial reproductive techniques: ovulation induction and in-vitro fertilization (IVF)

^b^Body mass index (BMI) ≥ 30 kg/m^2^

^c^Including chronic hypertension, gestational hypertension and preeclampsia

^c^Including pre-gestational and gestational diabetes mellitus

Table 2 summarizes selected pregnancy and perinatal outcomes among the different study groups. The mean offspring birthweight was highest in the NPL2 group. Following was the NPL1 and VD groups, and offspring born in elective CD had the lowest mean birthweight. Accordingly, the rate of LBW infants was highest in the elective CD group and lowest in the NPL2 group. Low Apgar scores were more prevalent in the CD groups compared with VD; however, offspring mean PH at birth was comparable between all groups. Rates of PPH were highest in the NPL2 group.

**Table 2:** Pregnancy and perinatal outcomes of the study population

Outcome	Vaginal delivery (n = 311,054)	NPL1 (n = 5149)	NPL2 (n = 2199)	Elective CD (n = 29,930)	P value
Gestational age (weeks ± SD)	39.1 ± 1.8	39.6 ± 1.5	39.6 ± 1.4	37.7 ± 2.3	< 0.001
Preterm birth ^a^, *n* (%)	17,477 (5.6)	179 (3.5)	43 (2.0)	5092 (17.0)	< 0.001
Birthweight (grams, mean ± SD)	3218 ± 495	3364 ± 495	3455 ± 448	3053 ± 625	< 0.001
Low birthweight ^b^, *n* (%)	17,971 (5.8)	235 (4.6)	34 (1.5)	4359 (14.6)	< 0.001
PH (± SD)	7.38 ± 1.25	7.30 ± 0.08	7.27 ± 0.09	7.33 ± 0.21	0.237
5 min Apgar < 7, *n* (%)	1140 (0.4)	69 (1.3)	55 (2.5)	496 (1.7)	< 0.001
Post-partum hemorrhage (%)	1867 (0.6)	15 (0.3)	18 (0.8)	139 (0.5)	< 0.001

^a^Delivery at gestational age < 37 weeks’ gestation

^b^Defined as birthweight < 2500 g

Selected infectious morbidities of the offspring in the different groups are shown in Table 3. Compared with VD, children among the CD groups were significantly more prone to suffer from ear-nose-throat (ENT), viral and respiratory infections. Total infectious-related hospitalization rates of the offspring were highest in the NPL1 and elective CD groups Apart from the major infectious categories mentioned above, other infection types (CNS, gastrointestinal, ophthalmic, dermatologic) demonstrated smaller absolute differences between groups and contributed less to the overall hospitalization trend.

**Table 3:** Selected infectious morbidities according to mode of delivery and indication for cesarean delivery

Infectious morbidity	Vaginal delivery (n = 311,054)	NPL1 (n = 5149)	NPL2 (n = 2199)	Elective CD (n = 29,930)	P value
Bacterial, n (%)	4480 (1.4)	87 (1.7)	22 (1.0)	493 (1.6)	0.008
Viral, n (%)	9759 (3.1)	206 (4.0)	87 (4.0)	1116 (3.7)	< 0.001
Respiratory, n (%)	42,231 (13.6)	749 (14.5)	308 (14.0)	4705 (15.7)	< 0.001
Central nervous system, n (%)	1596 (0.5)	23 (0.4)	7 (0.3)	183 (0.6)	0.081
Gastrointestinal, n (%)	9111 (2.9)	160 (3.1)	60 (2.7)	926 (3.1)	0.083
Ear–nose–throat (ENT), n (%)	19,620 (6.3)	395 (7.7)	146 (6.6)	2114 (7.1)	< 0.001
Ophthalmic, n (%)	3879 (1.2)	65 (1.3)	31 (1.4)	389 (1.3)	0.493
Dermatologic, n (%)	7246 (2.3)	138 (2.7)	51 (2.3)	660 (2.2)	0.217
**Total infectious-related hospitalizations (%)**	**73,953 (23.8)**	**1348 (26.2)**	**534 (24.3)**	**7829 (26.2)**	** < 0.001**

Bold values indicate the summary

**Fig. 1:**
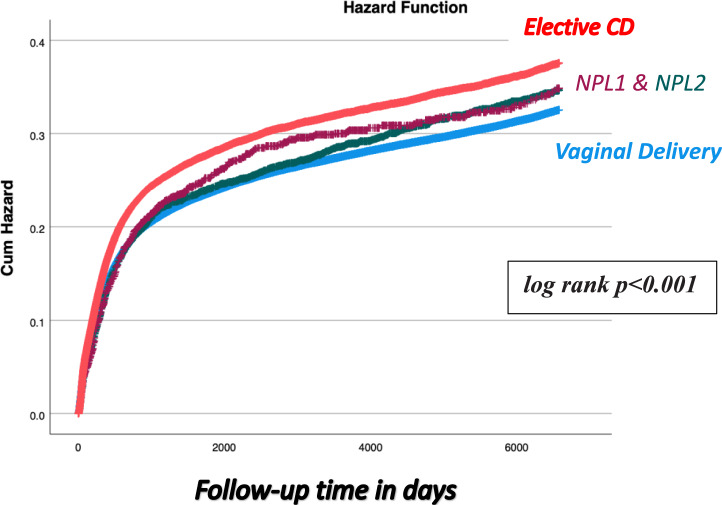
Kaplan–Meier survival curve demonstrating the cumulative incidence of infectious-related hospitalizations among study groups (Log-rank, *p* < 0.001)

A dose–response pattern was observed visually in the Kaplan–Meier curve (Fig. 1), with progressively higher cumulative incidence from VD → NPL2 → NPL1 → Elective CD (log-rank, *p* < 0.001). In the Cox regression model presented in Table 4 which controlled for maternal age, ethnicity, obesity and smoking, diabetes mellitus and hypertensive disorders, rupture of membranes, gestational age and the child’s birth year, elective CD and NPL1 groups were associated with an independent increased risk for offspring long-term infectious morbidity (aHR 1.13 [95% CI 1.11–1.15] and 1.10 [95% CI 1.05–1.16], respectively), while NPL2 showed comparable results compared to VD aHR 1.06 [95% CI 0.98–1.15]).

**Table 4:** Cox regression model for the association between mode of delivery and CD indication and offspring long-term infectious-related hospitalization risk

	Adjusted hazards ratio (aHR)	95% confidence interval	P value
Elective CD	**1.13**	**1.11–1.15**	** < 0.001**
CD for NPL1	**1.10**	**1.05–1.16**	** < 0.001**
CD for NPL2	**1.06**	**0.98–1.15**	**0.105**
Vaginal delivery	**1 (reference)**	**–**	**–**
Maternal age	0.98	0.98–0.99	< 0.001
Gestational age	0.96	0.95–0.96	< 0.001
Child birth year	1.01	1.01–1.01	< 0.001
Hypertensive disorders	1.03	1.01–1.06	0.019
Diabetes mellitus	1.11	1.08–1.14	< 0.001
Rupture of membranes	1.47	1.36–1.60	< 0.001
Smoking	1.48	1.39–1.58	< 0.001
Obesity	1.13	1.06–1.19	< 0.001
Ethnicity	0.90	0.89–0.92	< 0.001

Bold values indicate the dependent variables

## Discussion

### Principal findings

Our study suggests an essential role of maternal vaginal flora exposure during labor in shaping long-term infectious morbidity in children. We found that children born via vaginal delivery or cesarean delivery (CD) during the second stage of labor (NPL2) had fewer infectious-related hospitalizations compared to those born via CD in the first stage of labor (NPL1) or elective CD. The highest rates of infectious morbidity were observed in the elective CD group, where there was no exposure to maternal vaginal flora. These findings emphasize the importance of maternal vaginal flora in reducing long-term infectious risks, as lack of exposure to maternal vaginal flora during cesarean delivery may lead to a less diverse and potentially less resilient microbiome in offspring, increasing their risk for infections later in life.

## Results

We observed a significant association between the mode of delivery and subsequent infectious-related hospitalizations in children up to the age of 18 years and found that children who had the highest exposure to maternal vaginal flora (those born vaginally or in CD due to NPL2) had the lowest risk for long-term infectious morbidity as compared with those with partial (NPL1) or no exposure (elective CD) to maternal vaginal flora.

Our study validates previous research indicating that cesarean delivery, particularly when performed electively, is associated with an increased risk of infectious morbidity in offspring compared to vaginal delivery [8, 9]. This high risk is believed to be attributed to the absence of exposure to maternal vaginal flora, which plays a crucial role in the initial colonization of the neonatal gut microbiota and the development of the immune system [3].

The observed dose–response relationship between the mode of delivery and infectious morbidity also marks the importance of exposure to maternal flora even in circumstances of cesarean deliveries. Infants born via elective cesarean section, devoid of any exposure to maternal vaginal flora, exhibited the highest risk of infectious-related hospitalizations. In contrast, infants delivered by cesarean section for non-progressive labor in the second stage did not demonstrate a significantly elevated risk compared to vaginally delivered infants. This suggests that the critical period of exposure to maternal vaginal flora may occur earlier in labor and prior to delivery, presenting the importance of vaginal delivery when possible.

An interesting study by Leybovitz-Haleluya et al. [15] was aimed to investigate the long-term effects of CD due to non-reassuring fetal heart rate (NRFHR) on the risk of subsequent childhood infectious morbidity as compared with children born via CD for labor dystocia but with a normal FHR pattern. They found that being born via CD for NRFHR but not due to labor dystocia was a risk factor for long-term infectious morbidity of the offspring. However, they did not consider the stage of labor at which the fetus was delivered. In our study, we decided to exclude those born via CD due to fetal distress and thus we were able to better account for the stage of labor and not the fetal status at birth.

### Research implications

In contrast to our study, other research which aimed to investigate the association between cesarean delivery and the development of infectious diseases [16] found that children born by cesarean delivery did not have an increased risk of developing respiratory, gastrointestinal or urinary infections. Others [17, 18] found that cesarean delivery is associated with infectious hospitalization before, but not after, age 5 years. The study by Auger et al. [18] concluded that early childhood infectious morbidity was also present for operative vaginal delivery, which suggests that mechanisms other than exposure to maternal vaginal flora may explain the relationship. In light of the differences between our studies, future studies should explore the specific microbial profiles of offspring born via different delivery modes and their impact on immune system development and susceptibility to infections. In addition, interventions aimed at restoring or improving microbial transfer during cesarean deliveries, such as vaginal seeding or vaginal microbiota transfer (VMT) [13, 14, 19], warrant further investigation to determine their efficacy and safety in reducing the risk of infectious morbidity in the offspring.

### Strengths and limitations

The primary strengths of our study lie in its extensive sample size and prolonged duration of follow-up. As mentioned earlier, SUMC is the sole hospital in the Negev region, serving more than 1.3 million residents, so we anticipate that the majority of women who deliver at the institution would also seek pediatric care for their children if required. Thus, using our broad perinatal and pediatric databases, we were able to follow children from birth to 18 years of age and include various infectious morbidities that are common during infancy, childhood, and adolescence.

However, several limitations should be acknowledged. Our outcome assessment was based solely on hospitalization records from SUMC, which may underestimate the true burden of infectious morbidity, as milder community-treated infections were not captured. In addition, some children may have been hospitalized elsewhere, potentially leading to partial follow-up; nevertheless, as SUMC is the only tertiary hospital and main referral center in the region, we assume that most hospitalizations have occurred here, and loss to follow-up would likely affect all study groups.

As a retrospective study, some important variables that may also act as mediators or confounders were not available in our analysis. Information on intrapartum antibiotic exposure and breastfeeding practices was lacking, and socioeconomic status could not be directly assessed, though maternal ethnicity was used as a proxy given its correlation with sociodemographic background in the region. In addition, data regarding women who presented with pre-labor rupture of membranes (ROM) were included in the multivariable model, yet the duration and timing of ROM were unavailable, which may influence neonatal microbial exposure and contribute to residual confounding. To address these limitations, we used a multivariable regression model to carefully adjusted for many possible confounding factors including maternal characteristics and pregnancy complications and found it did not alter our findings.

To note, classification of first- and second-stage non-progressive labor was based on the diagnosis documented by the attending obstetrician, as original partograms were not consistently archived. Diagnostic criteria for labor dystocia may vary between providers and have evolved over time, introducing potential interobserver variability. In addition, the study spans nearly 3 decades, during which obstetric practices, neonatal care, vaccination policies, and antibiotic protocols changed substantially. This temporal heterogeneity may have influenced the outcomes. While the long study period is also a major strength, we acknowledge this limitation and included the child’s birth year in the multivariable model to account for some of the changes that might have occurred during this time.

Finally, as a single-center study conducted within a unique regional population, external generalizability may be limited. While SUMC serves an ethnically and socioeconomically diverse community, these characteristics are specific to the Negev and may not fully reflect other healthcare environments. Therefore, findings should be interpreted in the context of this setting.

## Conclusions

In an era characterized by increasing rates of cesarean sections and the consequent concern regarding their potential long-term health implications, our findings provide hypothesis-generating evidence that delivery mode and stage of labor may be associated with a higher risk of infectious-related hospitalizations in offspring. While our results suggest that reduced exposure to maternal vaginal flora—particularly in elective cesarean deliveries and cesarean performed during the first stage of labor—may be linked to higher long-term infectious morbidity compared with vaginal delivery or second-stage cesarean delivery, these observations arise from an observational study and do not establish causality. Residual confounding and the absence of direct microbiome validation limit the ability to draw definitive clinical recommendation.

Therefore, these findings should not be interpreted as a directive to alter delivery planning or prioritize vaginal delivery over elective cesarean section based solely on this work, and decisions must continue to consider maternal and neonatal risks associated with each mode of delivery.

This study highlights the need for future prospective and mechanistic research, ideally incorporating direct neonatal microbiome profiling, to validate these findings and further clarify the biological pathways linking labor exposure and long-term infectious outcomes.

## Supplementary Information

Below is the link to the electronic supplementary material.

Supplementary file1 (DOCX 52 KB)

## Data Availability

No datasets were generated or analysed during the current study.
